# Frequency of hereditary neuropathy with liability to pressure palsies (HNPP) due to 17p11.2 deletion in a Korean newborn population

**DOI:** 10.1186/s13023-018-0779-5

**Published:** 2018-03-15

**Authors:** Jong Eun Park, Seung-Jae Noh, Mijin Oh, Dae-Yeon Cho, So Young Kim, Chang-Seok Ki

**Affiliations:** 1 Department of Laboratory Medicine and Genetics, Samsung Medical Center, Sungkyunkwan University School of Medicine, Seoul, Republic of Korea; 2 LabGenomics Clinical Research Institute, LabGenomics, Seongnam, Gyeonggi-do Republic of Korea

**Keywords:** Hereditary neuropathy with liability to pressure palsies (HNPP), Prevalence, Korean population, Next-generation sequencing-based copy number variation analysis

## Abstract

Hereditary neuropathy with liability to pressure palsies (HNPP) is an autosomal dominant disorder mainly due to a deletion of chromosome 17p11.2 including *PMP22* (*PMP22* Del HNPP). The prevalence of HNPP is estimated to be 0.84 to 16 per 100,000, but could be underestimated because of the mild symptoms of HNPP. In this study, we estimated the prevalence of *PMP22* Del HNPP in a Korean newborn population who underwent next-generation sequencing (NGS)-based copy number variation (CNV) analysis. Of the 11,885 newborns tested by NGS-based CNV analysis, 17p11.2 deletions were found in seven samples. The prevalence of *PMP22* Del HNPP was estimated to be 58.9 per 100,000 (95% confidence interval (CI), 25.8–116.5) or 1 in 1698 (95% CI, 1/909–1/5000). Our data suggest that *PMP22* Del HNPP might not be uncommon at least in the Korean population.

Dear Editor,

Hereditary neuropathy with liability to pressure palsies (HNPP) is an autosomal dominant disorder. Approximately 80–90% of individuals with HNPP have a deletion of chromosome 17p11.2, which includes *PMP22* (*PMP22* Del HNPP). The remaining 10–20% have a pathogenic variant of *PMP22* [1–3]. The typical clinical symptoms of *PMP22* Del HNPP are the acute onset of non-painful focal sensory and motor neuropathy in the territory of a single nerve or brachial plexus [4]. Symptoms of *PMP22* Del HNPP occur mostly in the second or third decade, with a variable range from birth to the eighth decade. Several epidemiological studies reported that the prevalence of HNPP is 0.84 per 100,000 in the Republic of Ireland [5], 2.0 per 100,000 in Northern England [6], 7.3 per 100,000 in the city of Newcastle upon Tyne [6], and 16 per 100,000 in Southwestern Finland [7]. These studies estimated the prevalence of HNPP from specific populations who visited a hospital in a specific area. However, because the symptoms of HNPP can be mild and difficult to recognize, the true prevalence may have been underestimated in these studies.

The genetic diagnostic techniques for newborn screening have developed dramatically. Genetic testing that is able to detect abnormalities prior to the onset of symptoms in newborns can potentially reduce the risk of serious disease [8]. Copy number variation (CNV) of dose-sensitive genes and chromosome microdeletions play important roles in human genetic diseases. In Korea, the next-generation sequencing (NGS)-based CNV analysis has recently been introduced as an optional newborn screening program for early detection of sex chromosome aneuploidy and microdeletion syndromes, in which case early diagnosis may be beneficial to neonates. In this study, we estimated the prevalence of *PMP22* Del HNPP in Korean newborns that underwent NGS-based CNV analysis (EnfantGuard™, Labgenomics, Sungnam, Korea).

This study was approved by the local institutional review board. Between December 2015 and May 2017, a total of 11,885 neonates were evaluated for constitutional chromosomal abnormalities using the NGS-based CNV analysis. All parents agreed to this testing and provided informed consent. Capillary or cord blood was collected, and DNA was extracted using standard protocols. Libraries were prepared by a custom capture panel using a Customized Target Enrichment Kit (Celemics, Inc., Seoul, Korea). Paired-end sequencing was performed using the manufacturer’s instructions for Illumina NextSeq platform (Illumina, San Diego, CA, USA). More than six million sequence reads were analyzed using an in-house bioinformatics platform (CNABro™, Labgenomics, Sungnam, Korea) to detect more than one megabase-size CNV. Correlations between these diseases and the CNVs were evaluated by searching disease databases, including OMIM (https://www.omim.org), DECIPHER (https://decipher.sanger.ac.uk/), GeneReviews (https://www.ncbi.nlm.nih.gov/books/NBK1116/), and Orphanet (http://www.orpha.net/). A multiplex ligation-dependent probe amplification (MLPA) assay was performed using the SALSA MLPA KIT P033 CMT1 (MRC Holland, Amsterdam, The Netherlands) according to the manufacturer’s instructions to confirm the 17p deletion.

Of the 11,885 samples tested by NGS-based CNV analysis, 17p11.2 deletions were found in seven samples. All seven samples were from unrelated families and were collected within 7 days of birth. Six (85.7%) were female and three (42.9%) had a family history of HNPP. The deletion is 1.5 Mb in size, including the *PMP22* gene, which was confirmed by MLPA (Fig. 1). Therefore, the prevalence of *PMP22* Del HNPP in this population was estimated to be 58.9 per 100,000 [95% confidence interval (CI), 25.8–116.5] or 1 in 1698 (95% CI, 1/909–1/5000).

**Fig. 1:**
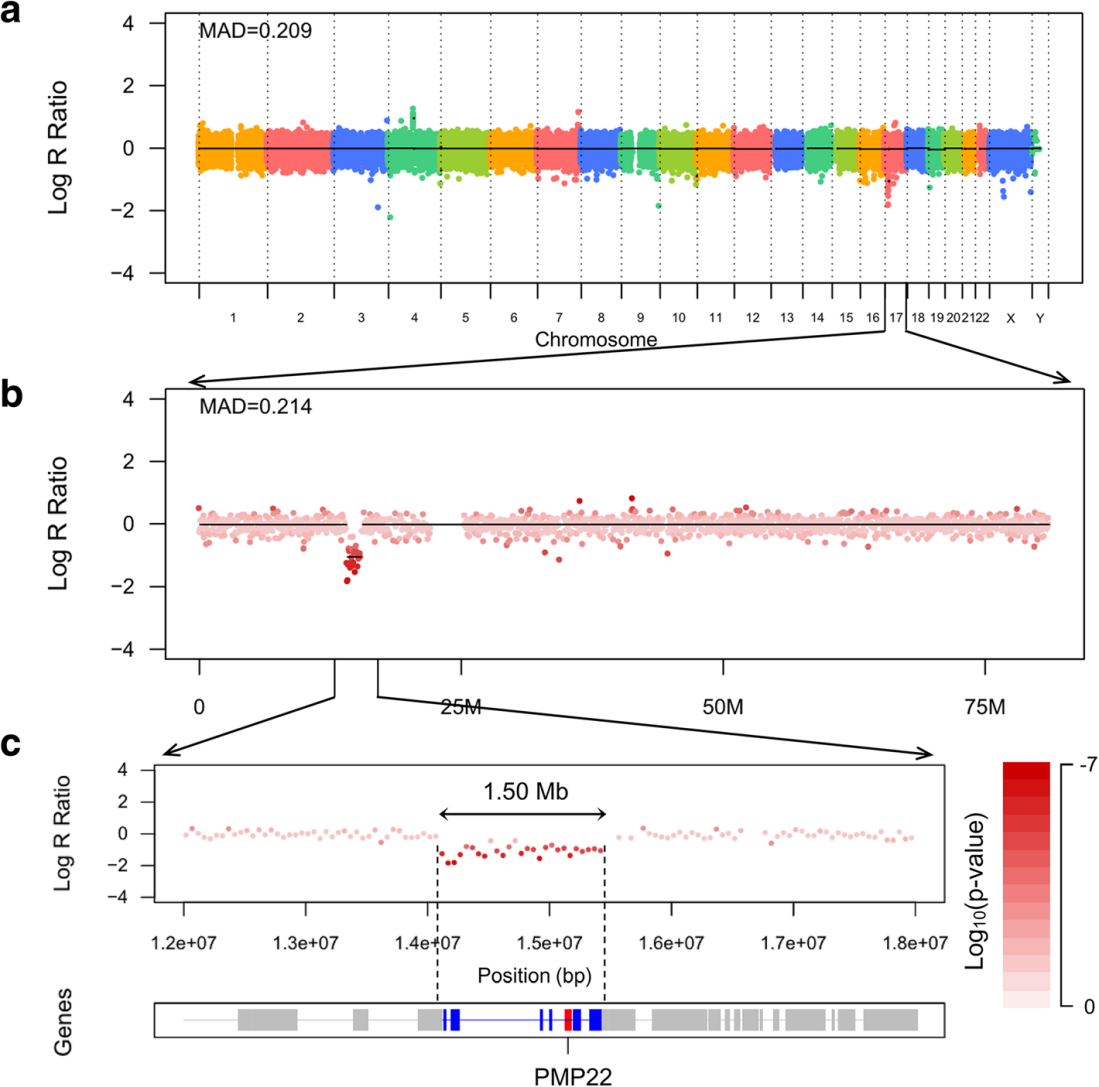
Combined log R ratio (LRR) plots of the 17p11.2 copy number variation (CNV) regions. **a** The panel shows a genome level LRR plot. **b** The panel shows the plot for chromosome 17. **c** The panel shows a detailed view of a CNV region in chromosome 17. Low values of LRR (less than − 1) indicate a deletion in the region of chromosome 17. MAD, median absolute deviation

The exact prevalence of HNPP remains unclear; however, a prior study from the Republic of Ireland and in southwestern Finland reported a prevalence of 0.84 per 100,000 to 16 per 100,000 [5–7]. However, in this study, the prevalence of HNPP was 58.9 per 100,000, which was three to seventy times higher than previous studies (Table 1). The difference between previous studies and our study is that we performed the tests on neonates before the onset of symptoms while the Irish, British, and Finnish studies were performed on symptomatic patients. Due to phenotypic variability or lack of clinical symptoms, many HNPP patients would not have necessarily known to visit a hospital. Therefore, the prevalence of HNPP may have been underestimated in previous studies. In addition, there may be differences in disease prevalence across studies due to differences among ethnic groups.

**Table 1:** Previously reported prevalence of hereditary neuropathy with liability to pressure palsies

Country	Number of cases	Estimated prevalence per 100,000	Year	Reference
Republic of Ireland	29	0.84	2017	Lefter et al. [5]
Northern England	59	2.0	2012	Foley et al. [6]
Newcastle upon Tyne	19	7.3	2012	Foley et al. [6]
South West Finland	69	16	1997	Meretoja et al. [7]
Korea	7	58.9	2017	This study

It is of note that there were 2 cases of *PMP22* duplication – a reciprocal phenomenon of *PMP22* deletion leading to Charcot-Marie-Tooth disease type 1A (CMT1A) – among 11,885 neonates so the estimated prevalence of *PMP22* duplication was 16.8 per 100,000 (95% CI, 2.8–55.6) or 1:5943 (95% CI, 1/1429–1/10000), which was comparable to the 1:3800–1:12,500 reported by van Paassen and colleagues [3]. However, the prevalence of *PMP22* duplication in our cohort could be underestimated because a significant proportion of parents with *PMP22* duplication already have CMT1A-related symptoms and may be more likely to request prenatal genetic diagnosis or preimplantation genetic diagnosis (PGD) rather than neonatal screening [9, 10].

Our study has several limitations. This study focused on asymptomatic infants. Most of all, the number of study subjects may not be enough to estimate the exact prevalence of rare genetic disorders. Considering that the symptoms of HNPP occur mostly in the second or third decade, the disease prevalence could be overestimated because some individuals with 17p11.2 deletion may not have HNPP phenotype in their life. Nevertheless, this study has the advantage of knowing the baseline frequency of 17p11.2 deletion causing *PMP22* Del HNPP. In conclusion, our data suggest that *PMP22* Del HNPP might not be uncommon at least in the Korean population.

## Abbreviations

CNV: Copy number variation
HNPP: Hereditary neuropathy with liability to pressure palsies
LRR: Log R ratio
MLPA: Multiplex ligation-dependent probe amplification
NGS: Next-generation sequencing
